# Supplementary material for: A rationally designed antimicrobial peptide from structural and functional insights of Clostridioides difficile translation initiation factor 1
Source: Microbiol Spectr. 2024 Feb 8;12(3):e02773-23. doi: 10.1128/spectrum.02773-23 (PMC10913371; doi:10.1128/spectrum.02773-23)
Supplement: Supplemental material — S1 (Structure comparison of CdIF1 and PaIF1), S2 (NMR measurement of CdIF1 binding to the 30S subunit), S3 (SEM data of CdIF1 peptide effect on E. coli cell membranes), and S4 (Cd-IF1 peptide inhibiting IPTG-induced GST expression in E. coli cells). [file spectrum.02773-23-s0001.docx]

**Supplementary Materials**

**A Rationally Designed Antimicrobial Peptide from Structural and Functional Insight of** ***Clostridioides difficile* Translation Initiation Factor 1**

Elvira Alanis ^1^, Faith Aguilar ^1^, Niaz Banaei ^2,3^, Frank B. Dean ^1^, Alexa Villarreal ^4^, Miguel Alanis ^1^, Karen Lozano ^4^, James M. Bullard ^1^, and Yonghong Zhang ^1,#^

^1^ School of Integrative Biological and Chemical Sciences, The University of Texas Rio Grande Valley, Edinburg, Texas, USA

^2^ Department of Pathology, Stanford University School of Medicine, Stanford, California; ^3^ Department of Medicine, Division of Infectious Diseases and Geographic Medicine, Stanford University School of Medicine, Stanford, California, USA.

^4^ Department of Mechanical Engineering, The University of Texas Rio Grande Valley, Edinburg, Texas, USA

^*^Correspondence to: [yonghong.zhang@utrgv.edu](mailto:yonghong.zhang@utrgv.edu)

**S1. Three-dimensional structure comparison between *C. difficile* IF1 and IF1 from *Pseudomonas aeruginosa*.**

The solution structure of *C. difficile* IF1 (Cd-IF1, PDB ID 6C00) is superimposed with IF1 from *Pseudomonas aeruginosa* (Pa-IF1, PDB ID 2N78) for structure comparison (**Figure S1**). Albeit the similarity of overall folding as a β-barrel, there are some notable differences in secondary structures, *i.e.*, three β-strands (β1, β3, β5). Two strands of Cd-IF1, β1 and β3, are longer than that of Pa-IF1, but β5 in Cd-IF1 is shorter than that of Pa-IF1. The long α-helix-containing loop is between β3 and β4, the longer β3 may suggest that this long helical loop in Cd-IF1 is less flexible than that of Pa-IF1. However, the shorter β5 at the C-terminal end of Cd-IF1 indicates a much longer unstructured tail, indicating the C-terminal tail of Cd-IF1 is more flexible than that of Pa-IF1. The structural difference may imply the distinct binding with their cognate 30S ribosomal subunit.


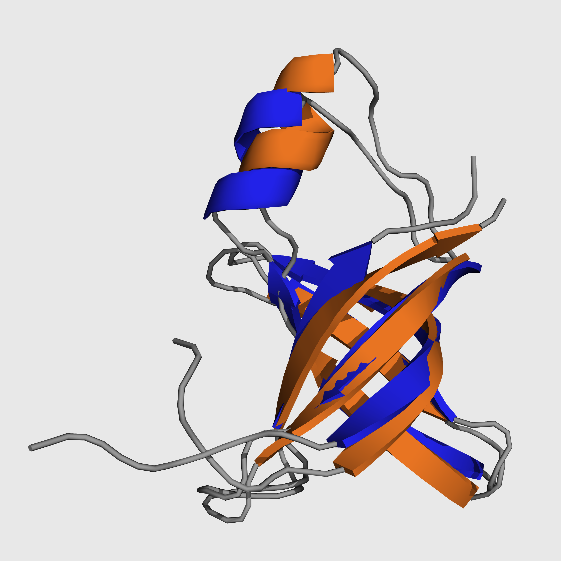

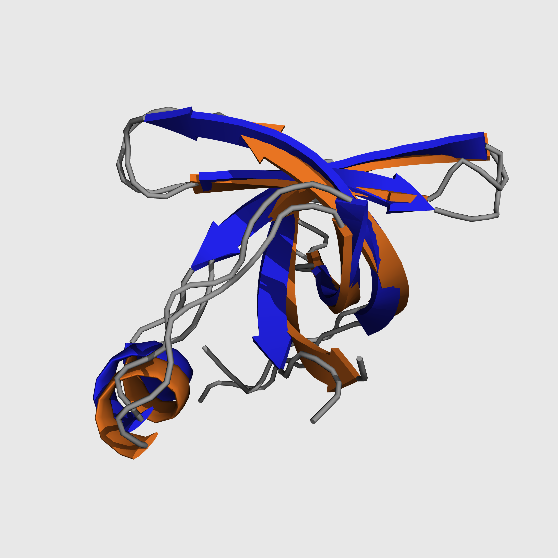


β5

β1

β2

β3

β4

β5

β4

β1

β3

β2

**Figure S1.** Structure superposition of *C. difficile* IF1 (secondary structure in blue, PDB ID 6C00) and IF1 from *P. aeruginosa* (secondary structure in orange, PDB ID 2N78). Five stranded β-sheets that form β-barrel structure are labeled.

**S2. NMR titration to estimate the apparent binding constant (Kd) of IF1 to the 30S subunit**

The ^15^N-labeled *Clostridioides difficile* IF1 (Cd-IF1, ~140 μM) proteins were titrated by adding a series of increasing amount of 30S subunits (200 μM) with 2D HSQC spectrum recorded in each titration. A total of four titration points in one set of experiment were performed with the molar ratios (30S: Cd-IF1) of 0.003, 0.009, 0.024, and 0.054. The relative intensity changes induced by the 30S were used to estimate the apparent dissociation constant (Kd) according to the equation below (**Eq. 1**). A series of changes of two representative amide peaks (M21 and H35) in the HSQC spectra induced by titrating the 30S ribosomal subunits were shown in **Figure S2 A**. The relative intensity changes and normalized chemical shift changes were plotted with the 30S ribosomal subunit concentration. The data were fitted to estimate the apparent dissociation constant (Kd) using [SciDAVis](http://scidavis.sourceforge.net/) program according to the equation (**Figure S2 B**):

$\boldsymbol{x}\mathbf{=}\frac{\mathbf{(}\boldsymbol{K}\mathbf{d+}\left[ \mathbf{T} \right]\boldsymbol{+[P])\pm}\sqrt{{\boldsymbol{(K}\mathbf{d+}\left[ \mathbf{T} \right]\mathbf{+[P]}\boldsymbol{)}}^{\mathbf{2}}\mathbf{-4[P][T]}}}{\mathbf{2}\boldsymbol{[P]}}$ **(Eq. 1)**

where $x$ is the relative peak intensity changes or normalized chemical shift changes, [T] and [P] are the concentrations of titrant (T) and protein (P). The fitting analysis with [T] and [P] treated as variables resulted in an apparent dissociation constant (*K*_d_) = 0.3±0.2 μM.


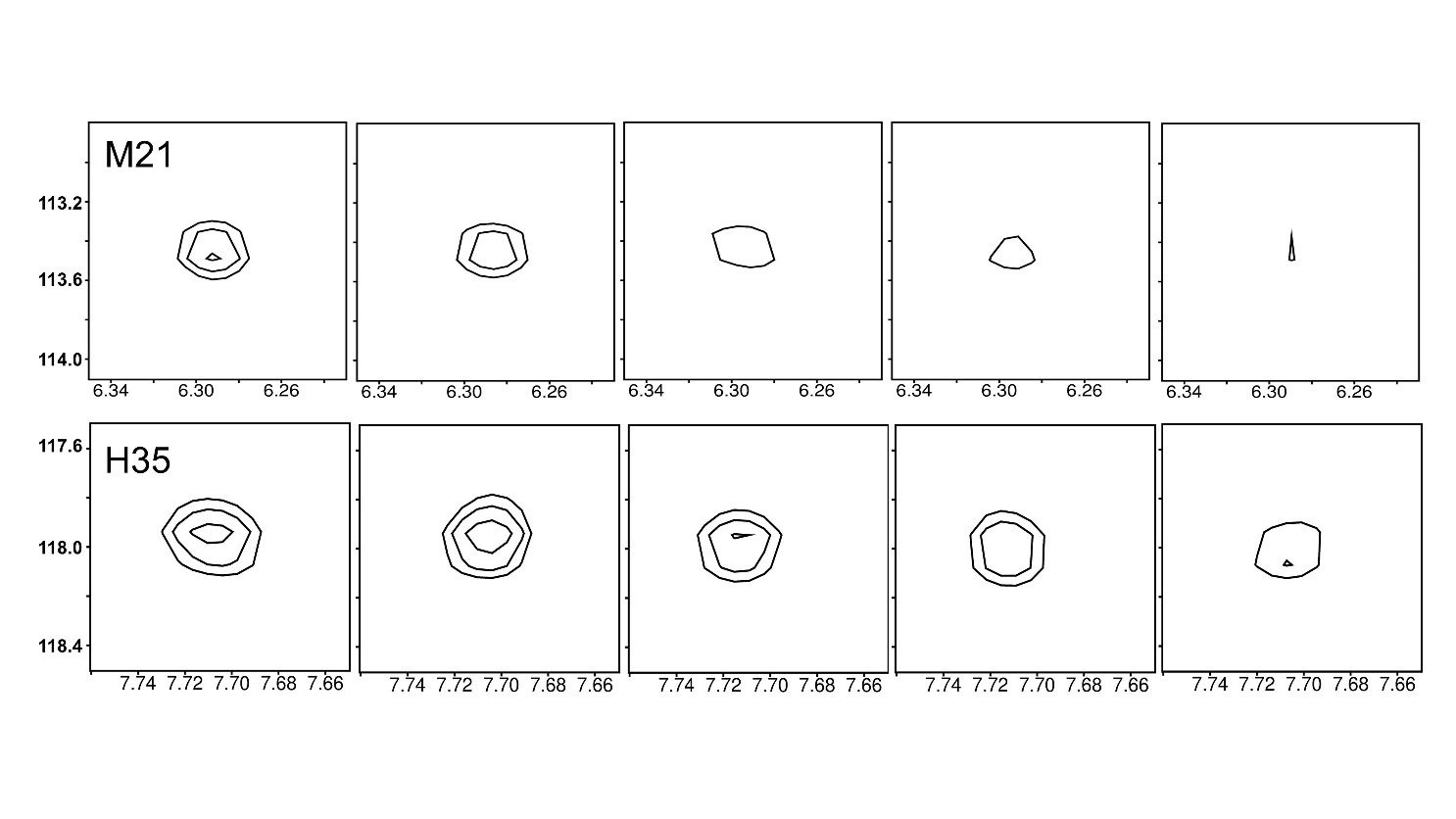


**Figure S2. A)** The changes of two HSQC peaks (M21 and H35) induced by various concentration of the 30S ribosomal subunit.


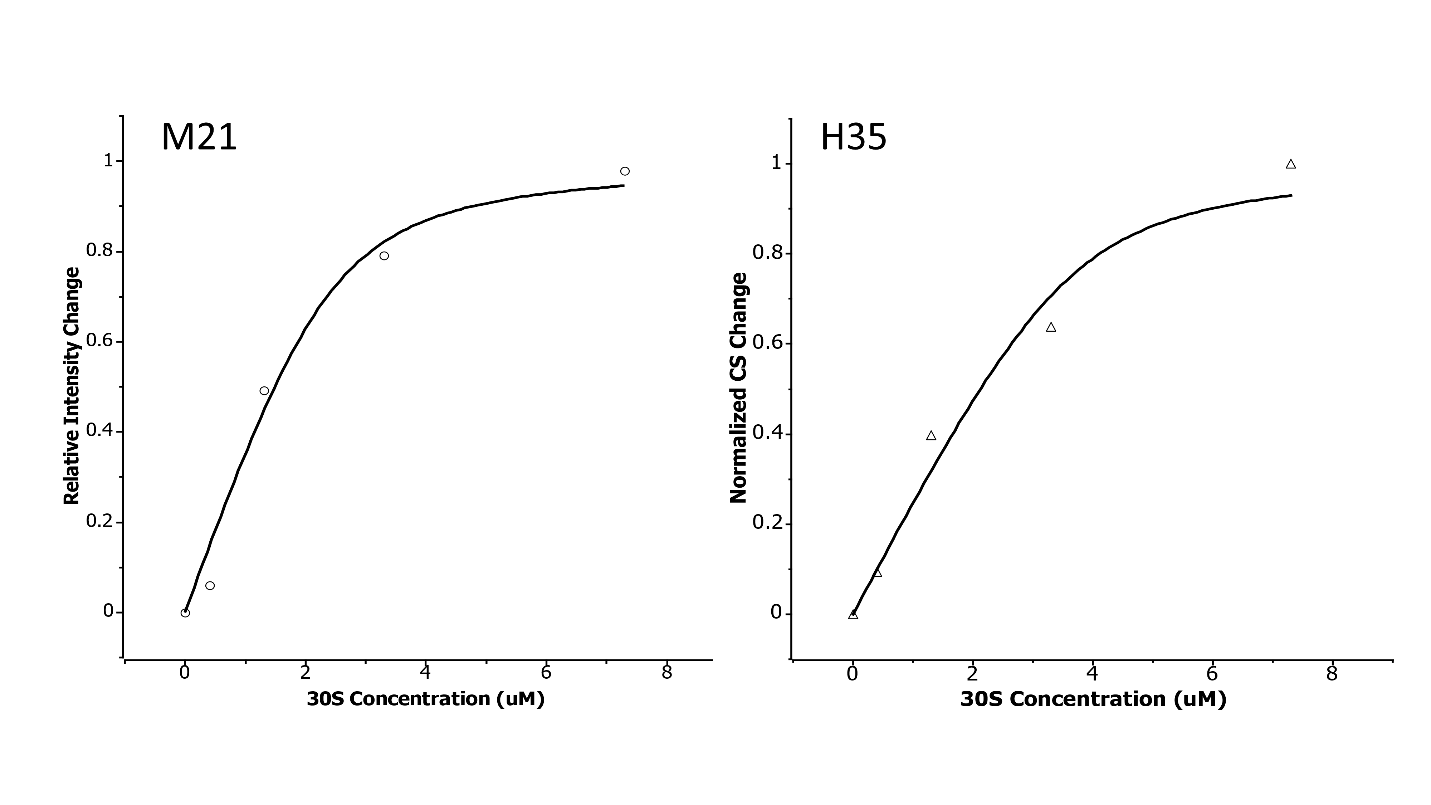


**Figure S2. B)** The relative changes of peak intensities (M21) and normalized changes of ^1^H and ^15^N chemical shifts (H35) were plotted against the concentration of the 30S ribosomal subunit and fitted.

**S3. Scanning electron microscope (SEM) characterization of Cd-IF1 peptide effect on bacterial cell membranes**

The effect of Cd-IF1 peptide on bacterial cell membranes was evaluated by a Field Emission Scanning Electron Microscope (Zeiss SIGMA VP-FESEM, Jena, Germany). *E. coli* (BL21(DE3) cells were grown in LB media at 37 °C to an optical density (A_600_) of 0.5. The cell culture was aliquoted to 3 tubes, two of which were treated with Cd-IF1 peptides (1× MIC) and Polymyxin B (1× MIC) and the third one was used as control (untreated). After 4-hour incubation at 37 °C, the cultures were spun down for 2 minutes at 6000 RPM to remove the supernatant. The cell pellets were washed by water three times to remove residual LB media. Finally, the cells were resuspended in 5 uL water and inoculated on the SEM pin stubs to dry out for SEM imaging.


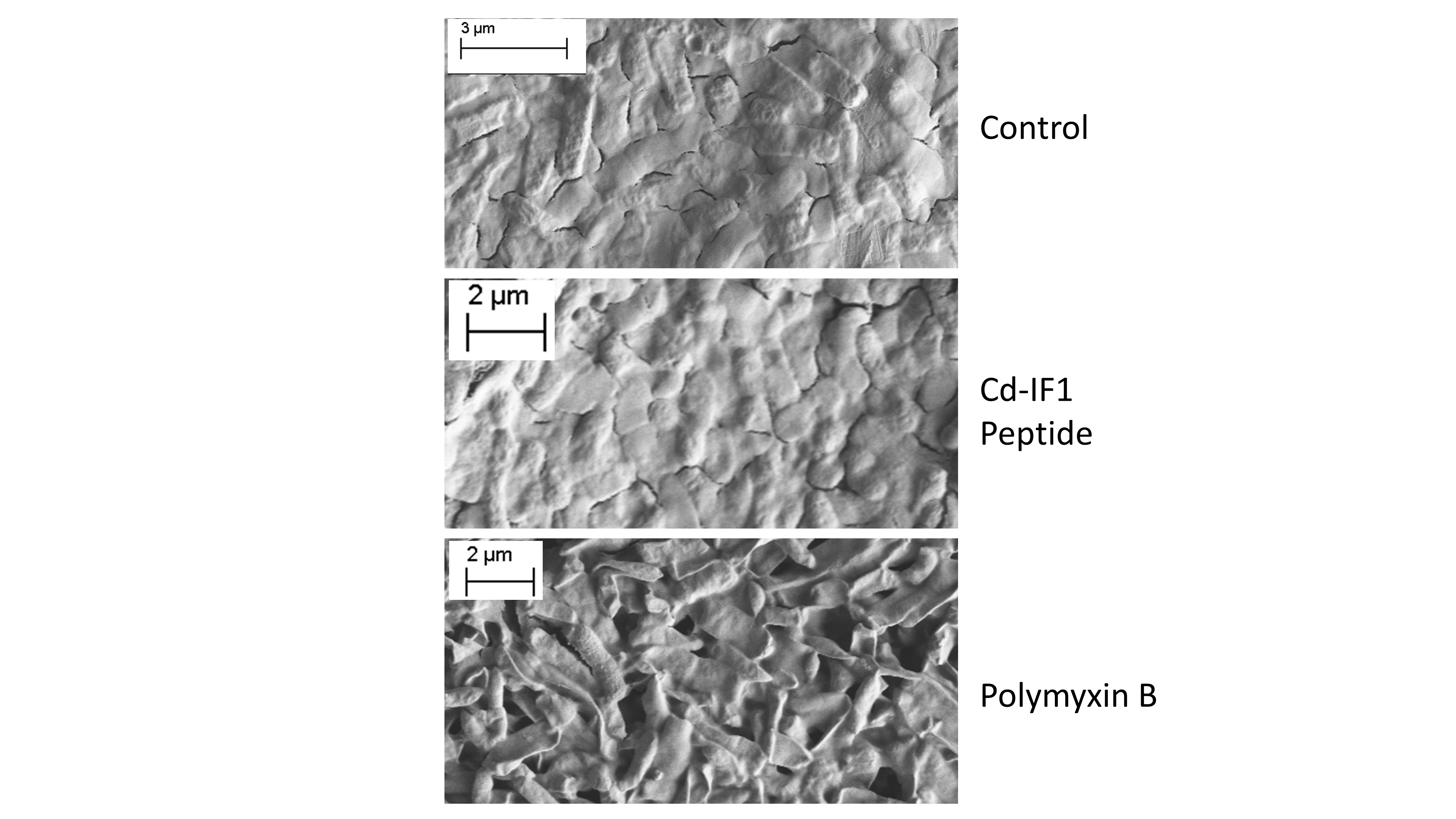


**Figure S3.** Scanning Electron Microscope (SEM) micrographs of *E. coli* untreated (top) and treated with Cd-IF1 peptide (1× MIC, middle) and Polymyxin B (1× MIC, bottom) for 4 hours.

**S4. Cd-IF1 peptide inhibiting IPTG-induced overexpression of GST protein in *E. coli* cells**

To evaluate if Cd-IF1 peptide can inhibit bacterial protein synthesis, a well-established and commonly used bacterial expression platform for recombinant protein production, was selected for IPTG-induced protein overexpression in the absence and presence of the peptide. The DNA plasmid, pGEX-4T-1 (a routine bacterial vector for expression of Glutathione *S*-Transferase (GST)-tagged fusion proteins (if a target gene is inserted in the vector); However, the vector plasmid in this study contains no other inserted gene) was transformed into BL21(DE3) competent cells. The cells were grown in LB media with ampicillin (pGEX-4T-1 contains ampicillin resistant gene) at 37 °C until OD600 reaching ~0.5. The cell cultures were aliquoted to small volumes for subsequent treatment. One culture was added with IPTG (Isopropyl ß-D-1-thiogalactopyranoside) to induce GST overexpression for 3.5 hours, while another was continuously incubated at the same condition for the same time period as protein expression control (without IPTG). Meanwhile, four more cultures were treated with Cd-IF1 peptides (1× MIC and 3× MIC), kanamycin (positive inhibition control), and DMSO (negative inhibition control as the peptide was dissolved in DMSO) for half an hour, and then IPTG was added into each cell culture for 3.5-hour induction time. Kanamycin was used as a positive inhibition control since it is a common aminoglycoside antibiotic and works by binding to the bacterial 30S ribosomal subunit, causing misreading of t-RNA, leaving the bacterium unable to synthesize proteins vital to its growth. All cultures were spun down to pellet cells. The cells were washed by water and subject to SDS-PAGE examination (**Figure S4**).


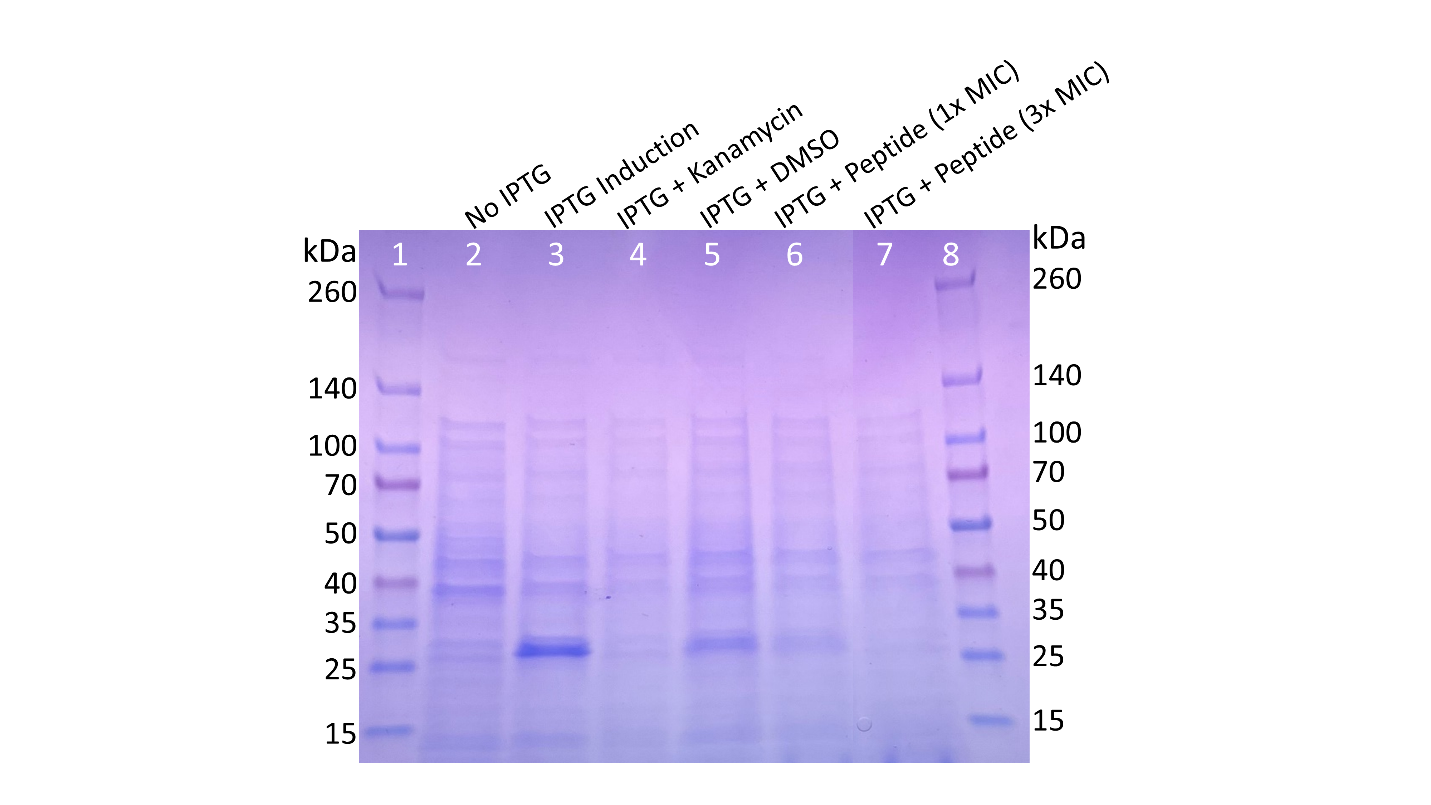


**Figure S4.** The SDS-PAGE gel of Cd-IF1 peptide inhibiting IPTG-induced GST protein (MW ~26 kDa) overexpression in *E. coli* cells. The BL21(DE3) cells harboring pGEX-4T-1 DNA plasmids were grown in LB media with ampicillin at 37 °C until OD600 reaching ~0.5. The cell cultures were treated with Cd-IF1 peptides (1× MIC and 3× MIC), kanamycin (positive inhibition control), and DMSO (negative inhibition control) for half an hour, and then IPTG was added into each cell culture for 3.5-hour induction time. The cells were washed by water and subject to SDS-PAGE examination in comparison with normal cell growth (without IPTG) and IPTG-induction (with IPTG) for 3.5 hours.
